# OMAMO: orthology-based alternative model organism selection

**DOI:** 10.1093/bioinformatics/btac163

**Published:** 2022-03-18

**Authors:** Alina Nicheperovich, Adrian M Altenhoff, Christophe Dessimoz, Sina Majidian

**Affiliations:** Department of Genetics, Evolution and Environment, University College London, London WC1E, UK; Department of Computer Science, ETH, 8092 Zurich, Switzerland; SIB Swiss Institute of Bioinformatics, 1015 Lausanne, Switzerland; SIB Swiss Institute of Bioinformatics, 1015 Lausanne, Switzerland; Department of Computational Biology, University of Lausanne, 1015 Lausanne, Switzerland; Department of Computer Science, University College London, London WC1E 6BT, UK; Department of Genetics , Evolution and Environment, University College London, London WC1E, UK; SIB Swiss Institute of Bioinformatics, 1015 Lausanne, Switzerland; Department of Computational Biology, University of Lausanne, 1015 Lausanne, Switzerland

## Abstract

**Summary:**

The conservation of pathways and genes across species has allowed scientists to use non-human model organisms to gain a deeper understanding of human biology. However, the use of traditional model systems such as mice, rats and zebrafish is costly, time-consuming and increasingly raises ethical concerns, which highlights the need to search for less complex model organisms. Existing tools only focus on the few well-studied model systems, most of which are complex animals. To address these issues, we have developed *O*rthologous *M*atrix and *A*lternative *M*odel *O*rganism (OMAMO), a software and a web service that provides the user with the best non-complex organism for research into a biological process of interest based on orthologous relationships between human and the species. The outputs provided by OMAMO were supported by a systematic literature review.

**Availability and implementation:**

https://omabrowser.org/omamo/, https://github.com/DessimozLab/omamo.

**Supplementary information:**

Supplementary data are available at *Bioinformatics* online.

## 1 Introduction

Model organisms are non-human species used in human biomedical research to study development, gene regulation and other cellular processes because they are relatively fast-growing, inexpensive and easy to manipulate. Most importantly, their use has been possible due to the evolutionary conservation of biological processes (Wangler *et al.*, 2017). Fast-moving progress in comparative genomics has allowed scientists to identify these evolutionary relationships by inferring human orthologs, genes that have diverged due to speciation (Fitch, 1970). Since orthologous genes tend to be functionally conserved and have common gene expression patterns, they are a better basis for model organism selection than other subtypes of homologs, which tend to functionally diverge faster (Altenhoff *et al.*, 2012; Zheng-Bradley *et al.*, 2010).

Currently used model organisms range from bacteria to complex mammals. The scientific community, however, aims to reduce the use of animals in research due to ethical implications, opting to use less complex organisms where possible. Currently available databases include MARRVEL (Wang *et al.*, 2019), the Alliance of Genome Resources portal (Alliance of Genome Resources Consortium, 2020), the Monarch Initiative (McMurry *et al.*, 2016) and MORPHIN (Hwang *et al.*, 2014). They focus on five to nine ‘traditional’ model organisms, most of which are complex organisms like mouse, rat and zebrafish. Moreover, their scope is restricted to human disease-related research. The only unicellular organisms considered in these databases are fission and budding yeast, whilst abundance of unicellular species in nature and their unique features make it difficult to find other non-complex model organisms for a biological process of interest.

To address the challenges above, we created an orthology-based *O*rthologous *M*atrix and *A*lternative *M*odel *O*rganism (OMAMO) database alongside a user-friendly web service. This database helps to select the best non-complex model organism for a biological process. Because the majority of species in the database have not been considered as model systems in the past, OMAMO has the potential to extend the set of organisms used in human biomedical research.

## 2 Materials and methods 

The OMAMO database is created using the OMAMO software that takes advantage of the OMA database of orthologous genes. For a given biological process, the output presents a list of potential model organisms ranked based on their orthologous relationships with human.

For each species, pyOMA library was used to extract human orthologs (Altenhoff *et al.*, 2021) (Supplementary Section S1.1). For each ortholog, pyOMA was used to retrieve Gene Ontology (GO) terms, which provide information about the gene product and can represent one of the following three aspects: molecular function, cellular component and biological process (Gene Ontology Consortium, 2021). Some GO terms are general (e.g. ‘cell division’), whilst others are more specific (‘G2/M transition of mitotic cycle’). To quantify the specificity of a GO term, we used information content (IC) calculated as *−*log(*p*), where *p* is its empirical frequency in the UniProt database (Pesquita, 2017). Thus, more specific GO terms have a higher IC value. The IC values were used to calculate GO-based functional similarity for each orthologous pair (Supplementary Section S1.2).

Orthologous pairs with GO-based functional similarity of <0.05 were discarded. This aims to reduce the number of orthologs that only share general GO terms in the output. Consequently, gene pairs from a given species were grouped according to the biological process GO term they share. To maintain sufficient specificity in functional similarity considered, only GO terms with IC ≥ 5 were kept. Finally, for each biological process GO term, species were ranked based on a scoring system, which takes into account the number of orthologs and average GO-based functional similarity across the genes relevant to the biological process. The higher the score, the more suitable an organism is for studying a process of interest.

**Fig. 1.**
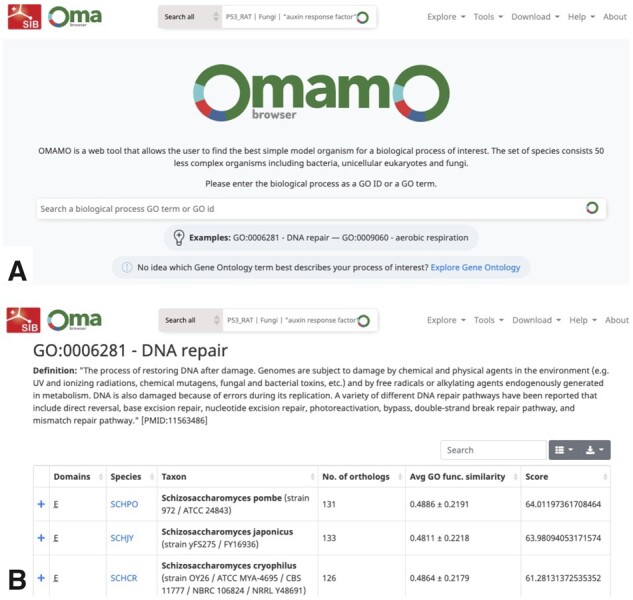
The web service interface. (**A**) The main browser page of OMAMO. The user can search a GO term (‘DNA repair’) or a GO ID (0006281). (**B**) The output page gives a list of species ranked based on the score, but the user has the option to sort the output based on the total number of orthologs or the average GO-based functional similarity by clicking on the up-down sorting icon. The user can view orthologs by clicking on the ‘+’ button.

We developed a freely accessible web service for OMAMO (Fig. 1), with the source code publically available. Out of the 50 species currently present in OMAMO, 31 are unicellular eukaryotes and the rest are bacteria. We suggest at least one model organism for 4620 out of 28 923 available biological process GO terms. Since OMAMO web service is integrated into the OMA website, OMAMO will be updated alongside OMA, meaning that the set of organisms will continue to grow and the OMAMO database will include the latest GO annotations.

To validate our results, we referred to experimental evidence through a systematic literature search on PubMed (Supplementary Section S2). The top five review articles on three of the well-studied organisms in OMA (*Dictyostelium* *discoideum*, *Neurospora* *crassa* and *Schizosaccharomyces* *pombe*) published in 2010–2021 were selected from the search output. Out of all biological processes which have been studied in one of the three organisms, the species of interest was in the top five model organism candidates in 42.6% of respective searches in OMAMO. This indicates that our algorithm is well supported by experimental data found in the literature.

## 3 Discussion

OMAMO is a freely available database and web service which aims to help scientists exploit alternative model species for human biomedical research. With the limited number of presently used model systems, the scientific community can now benefit from using other organisms, some of which could become model systems for processes that have previously only been studied in animals, leading to a reduction in their use in experimental research. Moreover, this is the first database that provides such a wide range of potential model organisms. Due to the lack of literature on using species presented in OMAMO, the validation of results proved to be challenging. The following step for output validation would be to utilize proposed model species as model systems in wet-lab experiments. In the future, we plan to greatly expand the species set and improve the scoring system by considering sequence similarity, conservation of protein structure and reproduction time. Additionally, we hope to provide unicellular model organisms based on their similarity to traditional model organisms like mouse and fruit fly. Besides, the search query could be extended to include gene names.

## Supplementary Material

btac163_Supplementary_Data
