# Supplementary material for: OMAMO: orthology-based alternative model organism selection
Source: Bioinformatics. 2022 Mar 18;38(10):2965–6. doi: 10.1093/bioinformatics/btac163 (PMC9113245; doi:10.1093/bioinformatics/btac163)
Supplement: btac163_Supplementary_Data [file btac163_supplementary_data.pdf]

Supplementary Information

1. Method

1.1 Finding orthologs

OMAMO identifies the most suitable model organisms based on their orthologous relationship to human. This is based on the fact that pairwise orthologs are useful when comparing two species (Zahn-Zabal et al. 2020). Since each of the 50 offered non-complex species is being individually compared to human, we opted to infer pairwise orthologs using a pre-existing python library pyOMA.

In more detail, the OMA algorithm first carries out an all-against-all comparison of amino acid sequences between all genes of two selected genomes. This generates a list of homologs. In order to filter out non-orthologous genes, the algorithm selects gene pairs with the highest reciprocal Smith-Waterman hits and closest evolutionary distances. Finally, pairs are verified by comparison with a third genome that acts as a 'witness of evolution' in case of asymmetric gene loss (Zahn-Zabal et al. 2020). For more detail about the OMA algorithm of orthology inference, please see methodology articles by Zahn-Zabal *et al.* and Roth *et al.*

1.2 The scoring system

In order to rank the model organism, we developed a scoring system based on the number of orthologs and average GO-based functional similarity across the genes relevant to the biological process. The score is calculated by adding up GO-based functional similarity (GFS) values for all orthologous gene pairs between human and the model organism (ModelGene_i and HumanGene_i):

$$score(\text{ModelOrganism}) = \sum_{i \in \text{orthologous pairs}} GFS(\text{ModelGene}_i, \text{HumanGene}_i) \quad (1)$$

GFS for each orthologous pair has been estimated using information content-based calculation of GO term overlap, which has been shown to be a good predictor of functional relatedness between genes (Mistry and Pavlidis, 2008). GO terms have a hierarchical structure, i.e. general GO terms are at the top, whilst more specific ('child') terms are found in the lower branches of the GO hierarchy. The OMA browser provides only more specific GO annotations, which were then propagated to the parental terms up until a term with information content of 5 and below was reached. This was done to avoid domain terms ('molecular function', 'cellular component', and 'biological function') and other very general terms that are shared amongst almost all orthologs. Taking these terms into account would have led to a skewed distribution of functional similarity.

For two orthologous genes G_1 and G_2, we gather a set of GO terms for each ortholog, namely GO_1 and GO_2. Then, the GO-based functional similarity is defined using the following equations:

$$GFS(G_1, G_2) = \frac{IC_{\cap}(\text{GO}_1, \text{GO}_2)}{IC_{\cup}(\text{GO}_1, \text{GO}_2)} \quad (2)$$

$$IC_{\cap}(\text{GO}_1, \text{GO}_2) = \sum_{\text{go}_i \in \text{GO}_1 \cap \text{GO}_2} IC(\text{go}_i) \quad (3)$$

$$IC_{\cup}(\text{GO}_1, \text{GO}_2) = \sum_{\text{go}_i \in \text{GO}_1 \cup \text{GO}_2} IC(\text{go}_i) \quad (4)$$

where GFS of two orthologous genes is based on Jaccard similarity (Popescu *et al.,* 2006). It is measured as the ratio of information content (IC) of overlapping GO terms (and their parents) to the union of information content stored by GO terms of both genes (and their parental terms).

1.3 Threshold settings

If the user chooses to use the website interface for their research, their result will be given with the following three filters set to default settings: (i) Minimal functional similarity of orthologous pairs (≥ 0.05) (Supplementary Figure 1); (ii) Minimum number of orthologous pairs per biological process (no threshold) (Supplementary Figure 2); (iii) Minimum IC value of GO terms (≥ 5) (Supplementary figure 3). However, if the user opts to use the software, they can change these settings according to their needs (e.g. if they wish to only consider orthologs with functional similarity of above 0.5 or if they only want to see species that have more than a certain number of orthologs for a given biological process).

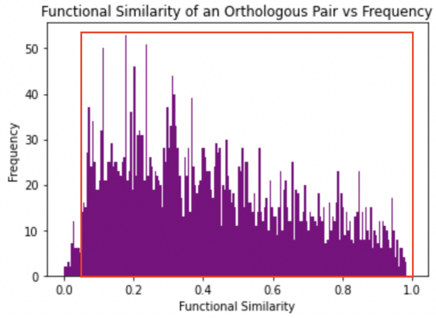

Supplementary Figure 1. Distribution of functional similarity across orthologous pairs between *Dictyostelium discoideum* and *Homo Sapiens*. The red box shows default settings, i.e. the database only includes those orthologous pairs that have functional similarity of above 0.05. If the user wishes to only see outputs only for orthologs with high functional similarity, they can choose the threshold value to be higher.

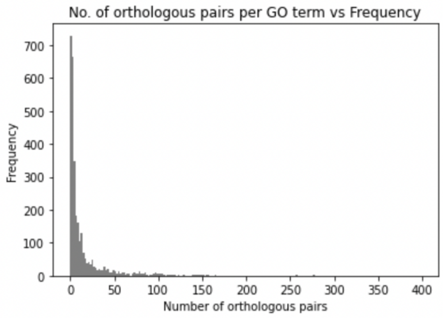

Supplementary Figure 2. This histogram demonstrates distribution of orthologous pairs across biological process GO terms. For example, the peak to the left demonstrates that there are over 700 GO terms that have only one ortholog from *Dictyostelium discoideum* associated with them.

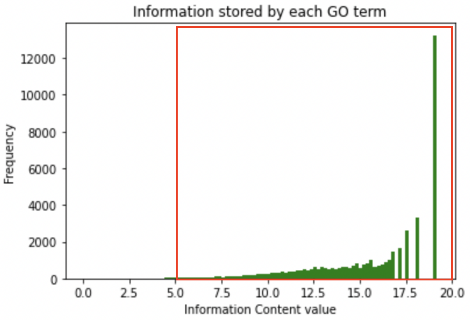

Supplementary Figure 3. Distribution of Information content across all GO terms found in the UniProt as of July 2020. The red box shows the default setting of information content ≥ 5.

For example, if the user searches GO ID 0010737 ('protein kinase A signalling') with default settings, the output that they would get is like that shown in Supplementary Figure 4 (A). However, if the user changes the lower threshold for the number of orthologs in a model organism from 0 (default) to 4, their output would be different, as shown in Supplementary Figure 4 (B).

(A)

	Species	No. of OGs	Average func. similarity ± st. dev.	Score
1	SORMK	4	0.2868 ± 0.1252	1.15
2	NEUT9	4	0.2868 ± 0.1252	1.15
3	EMEND	4	0.2415 ± 0.1445	0.97
4	THETO	3	0.2749 ± 0.16	0.82
5	EMENI	4	0.2051 ± 0.146	0.82
6	SCHJY	4	0.1783 ± 0.0911	0.71
7	DICPU	1	0.4542 ± 0.0	0.45
8	DICDI	1	0.4542 ± 0.0	0.45
9	NEUCR	1	0.4368 ± 0.0	0.44
10	SCHCR	1	0.3092 ± 0.0	0.31
11	SCHOY	1	0.3092 ± 0.0	0.31
12	SCHPO	1	0.2929 ± 0.0	0.29

(B)

	Species	No. of OGs	Average func. similarity ± st. dev.	Score
1	SORMK	4	0.2868 ± 0.1252	1.15
2	NEUT9	4	0.2868 ± 0.1252	1.15
3	EMEND	4	0.2415 ± 0.1445	0.97
4	EMENI	4	0.2051 ± 0.146	0.82
5	SCHJY	4	0.1783 ± 0.0911	0.71

Supplementary Figure 4. (A) Output for 0010737 with default settings. (B) Output for 0010737 where the lower threshold for number of orthologs has been set to 4.

Additionally, when using the code, the user can pick any combination of species present in OMA.

2. Systematic literature search

We chose 15 most relevant publications for the string ((species name) AND (model organism) AND (human)) in PubMed, five for each of the three species *(Dictyostelium discoideum, Neurospora crassa,* and *Schizosaccharomyces pombe),* from which we collected a total of 94 terms describing a biological process that has been studied in the species of interest. Searching for 21 of these gave no output or the species of interest wasn't in the list of suggested model organisms, which is denoted as '-' in the table below. In 40 out of 94 searches (42.6%), the species of interest was ranked as number 1-5. In 57 out of 94 searches (60.6%), the organism was in the top 10 suggested model systems, as summarised in the Supplementary Table 1 below.

Supplementary Table 1

Organism	Biological GO Terms (Rank No.)
Dictyostelium discoideum (Bozzaro, 2019; Pearce *et al.*, 2019; McLaren *et al.*, 2019; Martín-González *et al.*, 2021; Stuelten *et al.*, 2018)	Cell motility (6), chemotaxis (1), phagocytosis (1), macropinocytosis (-), cell adhesion (2), programmed cell death (4), autophagy (1), cytokinesis (14), lysosome organisation (2), cell-substrate adhesion (1), chemotaxis to cAMP (-), establishment of cell polarity (8), cell migration (1), phototaxis (-), mitochondrial transcription (1), protein insertion into mitochondrial outer membrane (-), aerobic respiration (2), mitochondrial organisation (10), mitochondrial localisation (11), thermotaxis(-), oxidative phosphorylation (14), reactive oxygen species biosynthetic process (1), mitochondrial calcium ion homeostasis (4), mitochondrial fission (19), macroautophagy (2), cellular water homeostasis (3), actin cytoskeleton organisation (2), protein phosphorylation (9), small GTPase-mediated signal transduction (2), multivesicular body assembly(-), multivesicular body organization (-), endosomal transport (2), vacuole organization (2), vacuolar transport (4), actin polymerisation and depolymerisation (2), regulation of cell-substrate adhesion (2), cellular component assembly (-), vesicle-mediated transport (3), intracellular signal transduction (3), regulation of signal transduction (11), regulation of intracellular signal transduction (3), protein processing (12), Notch receptor processing (-), amyloid precursor protein catabolic process (-), membrane protein intracellular domain proteolysis (1), ephrin receptor signalling pathway (-), organelle assembly (15), endosomal transport (2),

	multivesicular body assembly (-), energy derivation by oxidation of organic compounds (2), mitochondrial respiratory chain complex I assembly (6), mitochondrial electron transport NADH to ubiquinone (7), cellular protein complex assembly (15), mitochondrial ATP synthesis coupled electron transport (17), anion transport (4), regulation of biological quality (-).
Neurospora crassa (Jolma *et al.*, 2010*;* Ridenour *et al.*, 2020; Pelham *et al.*, 2020; Dunlap and Loros, 2017; Hevia *et al.*, 2016)	Circadian rhythm (-), histone methylation (19), histone H3 K27 methylation (-).
Schizosaccharomyces pombe (Allshire and Madhani, 2018; Matthews and Vosshall, 2020; Lin and Austriaco, 2014; Zhao, 2017; Florea, 2017)	Heterochromatin organisation (12), heterochromatin assembly (16), histone methylation H3K9 (-), gene silencing by RNA (5), cell division (2), programmed cell death(11), ageing (9), autophagy (9), necrotic cell death(-), intrinsic apoptotic signalling pathway (-), apoptotic process (6), regulation of cell death (4), mRNA splicing via spliceosome (6), RNA interference (8), mitochondrial inheritance (-), TOR signalling (4), response to reactive oxygen species (3), vacuolar acidification (10), mitochondrial fission (4), GMP biosynthesis (23), regulation of mitochondrial membrane potential (3), apoptotic DNA fragmentation (5), nuclear fragmentation involved in apoptotic nuclear change (-), reactive oxygen species process biosynthetic process (10), cell death (11), NAD+ biosynthetic process (16), response to virus (1), regulation of cell cycle (5), regulation of vesicle-mediated transport (11), DNA replication (8), DNA repair (1), G2/M transition of mitotic cycle (2), mRNA processing (8), response to hydrogen peroxide (3), response to heat (8).

Supplementary References

Allshire,R.C. and Madhani,H.D. (2018) Ten principles of heterochromatin formation and function. *Nat. Rev. Mol. Cell Biol.*, **19**, 229–244.

Bozzaro,S. (2019) The past, present and future of Dictyostelium as a model system. *Int. J. Dev. Biol.*, **63**, 321–331.

Dunlap,J.C. and Loros,J.J. (2017) Making Time: Conservation of Biological Clocks from Fungi to Animals. *Microbiol Spectr*, **5**.

Florea,M. (2017) Aging and immortality in unicellular species. *Mech. Ageing Dev.*, **167**, 5–15.

Jolma,I.W. *et al.* (2010) Circadian oscillators in eukaryotes. *Wiley Interdiscip. Rev. Syst. Biol. Med.*, **2**, 533–549.

Hevia,M.A. *et al.* (2016) Circadian clocks and the regulation of virulence in fungi: Getting up to speed. *Semin. Cell Dev. Biol.*, **57**, 147–155.

Lin,S.-J. and Austriaco,N. (2014) Aging and cell death in the other yeasts, Schizosaccharomyces pombe and Candida albicans. *FEMS Yeast Res.*, **14**, 119–135.

Pearce,X.G. *et al.* (2019) The Dictyostelium model for mitochondrial biology and disease. *Int. J. Dev. Biol.*, **63**, 497–508.

Martín-González,J. *et al.* (2021) Dictyostelium discoideum as a non-mammalian biomedical model. *Microb. Biotechnol.*, **14**, 111–125.

Matthews,B.J. and Vosshall,L.B. (2020) How to turn an organism into a model organism in 10 'easy' steps. *J. Exp. Biol.*, **223**.

McLaren,M.D. *et al.* (2019) Recent Insights into NCL Protein Function Using the Model Organism Dictyostelium discoideum. *Cells*, **8**.

Mistry, Meeta, and Paul Pavlidis. 2008. “Gene Ontology Term Overlap as a Measure of Gene Functional Similarity.” *BMC Bioinformatics* 9 (August): 327.

Pelham,J.F. *et al.* (2020) Intrinsic disorder is an essential characteristic of components in the conserved circadian circuit. *Cell Commun. Signal.*, **18**, 181.

Popescu, Mihail, James M. Keller, and Joyce A. Mitchell. 2006. “Fuzzy Measures on the Gene Ontology for Gene Product Similarity.” *IEEE/ACM Transactions on Computational Biology and Bioinformatics / IEEE, ACM* 3 (3): 263–74.

Ridenour,J.B. *et al.* (2020) Polycomb Repression without Bristles: Facultative Heterochromatin and Genome Stability in Fungi. *Genes* , **11**.

Roth,A.C. *et al.* (2008) Algorithm of OMA for large-scale orthology inference. *BMC Bioinformatics*, **9**, 518.

Stuelten,C.H. *et al.* (2018) Cell motility in cancer invasion and metastasis: insights from simple model organisms. *Nat. Rev. Cancer*, **18**, 296–312.

Zhao,R.Y. (2017) Yeast for virus research. *Microb. Cell Fact.*, **4**, 311–330.
